# Co‐mutational assessment of circulating tumour DNA (ctDNA) during osimertinib treatment for T790M mutant lung cancer

**DOI:** 10.1111/jcmm.14565

**Published:** 2019-08-08

**Authors:** Puyuan Xing, Xiaohong Han, Sha Wang, Yutao Liu, Sheng Yang, Xuezhi Hao, Yan Wang, Peng Liu, Junling Li, Lin Wang, Lianpeng Chang, Yanfang Guan, Zhishang Zhang, Di Wu, Jiarui Yao, Xin Yi, Yuankai Shi

**Affiliations:** ^1^ Department of Medical Oncology, Beijing Key Laboratory of Clinical Study on Anticancer Molecular Targeted Drugs, National Cancer Center/National Clinical Research Center for Cancer/Cancer Hospital Chinese Academy of Medical Sciences and Peking Union Medical College Beijing China; ^2^ Department of Clinical Laboratory, National Cancer Center/Cancer Hospital Chinese Academy of Medical Sciences and Peking Union Medical College Beijing China; ^3^ Geneplus‐Beijing Beijing China

**Keywords:** circulating tumour DNA, non‐small‐cell lung cancer, osimertinib, resistance

## Abstract

Osimertinib is designed to target the secondary resistant EGFR T790M mutant and has shown outstanding clinical efficacy. However, the prognostic prediction of osimertinib patients is a big problem in clinical practice. The resistance mechanism of osimertinib is also not fully understood. NGS and a 1021 gene capture panel were used to analyse the somatic mutation profile of thirty‐six lung adenocarcinoma patients' serial ctDNA samples. Progression‐free survival of subgroup patients is analysed. Patients harbour TP53 mutations and patients with higher TMB value in pre‐treatment samples showed a shorter PFS. Moreover, compared to CT evaluation, ctDNA changes generally correlated with treatment responses in most patients. Novel resistance mechanisms are discovered including EGFR mutations and alternative activation pathway. Our results implied a broad potential of ctDNA as an adjuvant tool in practical clinical management of NSCLC patients. ctDNA could help with clinical practice during osimertinib treatment, regarding monitoring tumour response, detecting development of heterogeneity, identifying potential resistance mechanisms, predicting treatment efficacy and patient outcome.

## 1 INTRODUCTION

Tissue biopsy is the gold standard for the analysis of prognostic and predictive biomarkers.^1^ However, in practice, tissue sampling is sometimes not feasible^2^ and liquid biopsy with circulating tumour DNA (ctDNA) analysis by next‐generation sequencing (NGS) can provide a viable alternative. Liquid biopsy based on ctDNA has become a reliable and convenient tool for the comprehensive genomic assessment of non‐small‐cell lung cancer (NSCLC) patients for identifying actionable mutations as well as co‐mutations and acquired resistance alterations.^3^, ^4^, ^5^ In recent clinical trials of immunotherapy for advanced NSCLC patients, TMB based on plasma ctDNA also exhibited good performance as a prognostic biomarker.^6^

Osimertinib is an irreversible third‐generation EGFR‐TKI targeting the secondary resistant EGFR T790M mutant with outstanding clinical efficacy.^7^, ^8^ Unfortunately, acquired resistance still occurs after a median of 10 months^4^ with various resistance mechanisms.^9^, ^10^, ^11^, ^12^, ^13^ Moreover, emerging data indicated the co‐occurrence of TP53 mutations is associated with shortened progression‐free survival.^14^, ^15^, ^16^ Identifying other acquired mutations during treatment could allow better prognostication and therapeutic strategies.

In this study, we enrolled thirty‐six EGFR‐mutant advanced lung adenocarcinoma patients, who were confirmed T790M‐positive based on tumour tissue genotyping assay, and investigated the clinical implications of serial analysis using a 1021 gene capture panel of ctDNA during osimertinib treatment. Our results implied a broad potential of ctDNA as an adjuvant tool in practical clinical management.

## 2 MATERIALS AND METHODS

### 2.1 Study population

A total of 36 metastatic lung adenocarcinoma patients with acquired EGFR T790M mutations were enrolled from National Cancer Center/Cancer Hospital, Chinese Academy of Medical Sciences and Peking Union Medical College. The study was reviewed and approved by Ethics Committee of this institution. All patients provided written contents according to ethical regulations. For each patient, a volume of 10 mL blood was collected before osimertinib treatment, at individual response assessment time‐point and after disease progression. Response assessments were performed every 3 months during treatment. Progression‐free survival (PFS) was measured from the beginning of osimertinib treatment to progression confirmed by computer tomography (CT) scan according to RECIST 1.1,^17^ or treatment cessation, or death.

### 2.2 Targeted NGS and data processing

cfDNA extraction and library construction were performed according to previous publication.^18^, ^19^ Peripheral blood lymphocytes (PBLs) from the first centrifugation were used for the extraction of germline genomic DNA. Target enrichment was performed with a custom SeqCap EZ Library (Integrated DNA Technologies, IDT). The capture probe was designed based on ~1.5 Mb genomic regions of 1021 genes frequently mutated in NSCLC and other common solid tumours. Capture hybridization was carried out according to the manufacturer's protocol. Following hybrid selection, the captured DNA fragments were amplified and then pooled to generate several multiplex libraries. Sequencing was carried out using Illumina 2 × 75 bp paired‐end reads on an Illumina HiSeq 3000 instrument according to the manufacturer's recommendations using TruSeq PE Cluster Generation Kit v3 and the TruSeq SBS Kit v3 (Illumina).

After the removal of terminal adaptor sequences and low‐quality data, reads were mapped to the reference human genome (hg19) and aligned using BWA (0.7.12‐r1039). MuTect2 (3.4‐46‐gbc02625) was employed to call somatic small insertions and deletions (InDels) and single nucleotide variants (SNVs). Contra (2.0.8) was used to detect copy number variations. All final candidate variants were manually verified with the integrative genomics viewer browser.

### 2.3 Pyclone and TMB analysis

PyClone was used to analyse the clonal population structure of ctDNA collected serially from each patient.^20^ The copy number information of each single nucleotide variation (SNV) was used as input. Variants located in the cluster with greatest mean cancer cell fraction (CCF) were defined as clonal and the rest were subclonal. TMB of blood (bTMB) was analysed using SNVs (non‐synonymous only) and indels at allele frequencies of ≥0.5%. The cut‐off value for bTMB‐H and bTMB‐L is defined as 9 mutations/Mb, approximately the median value in our assay experience.

### 2.4 Statistical analysis

Survival analysis was performed by multivariate Cox proportional hazards regression analysis and Kaplan‐Meier survival analysis with log‐rank test. IBM SPSS software (V23.0) and GraphPad Prism (V6.01) were used. All tests were two‐sided and considered statistically significant at *P* < .05.

## 3 RESULTS

### 3.1 **Patients**'** characteristics**

Before starting osimertinib, a tumour tissue DNA PCR assay was performed to ascertain the EGFR genotyping. Thirty‐six T790M‐positive patients were enrolled. Twenty‐four patients (67%) harboured exon 19 deletion, 11 (31%) harboured L858R, and one patient was detected to have an exon 20 mutation S768I. Patient clinical characteristics are listed in Table 1. The average age was 63.4 (44‐79) years, and 32 (89%) were non‐smokers. Before treated with osimertinib, 15 (42%) had received only first‐generation EGFR‐TKI; 2 (5%) had only chemotherapy; and 19 (53%) had been previously exposed to both first‐generation EGFR‐TKI and chemotherapy.

**Table 1:** Clinical characteristics of all patients

Characteristics	Patients (n = 36)
Age	
Mean	63.4
Range	44‐79
Gender (%)	
Male	12
Female	24
Smoking status	
Non‐smoker	32
Current‐smoker	4
Previous lines of therapy	
0	0
1	19
2	11
3	5
Treatment before osimertinib	
Gefitinib/Erlotinib/lcotinib	15
Chemotherapy	2
Gefitinib/Erlotinib/lcotinib + chemotherapy	19
Treatment‐native	0
EGFR mutation status	
Exon 19 deletion	24
L858R	11
Exon 20 mutation	1

We evaluated these patients every 6 weeks until disease progression or death. Efficacy assessment was evaluated by RECIST 1.1.^17^ During the treatment period, 34 patients had at least one confirmed partial response (PR); only one patient had progressive disease (PD) at first response assessment; and 4 patients died before PD. Objective response rate (ORR) of osimertinib was 97.2%. At the time of data cut‐off, a total of 17 (47.2%) patients experienced PD or died. Overall survival (OS) data are not yet mature.

### 3.2 Pre‐treatment ctDNA features correlate with progression‐free survival

**Figure 1:**
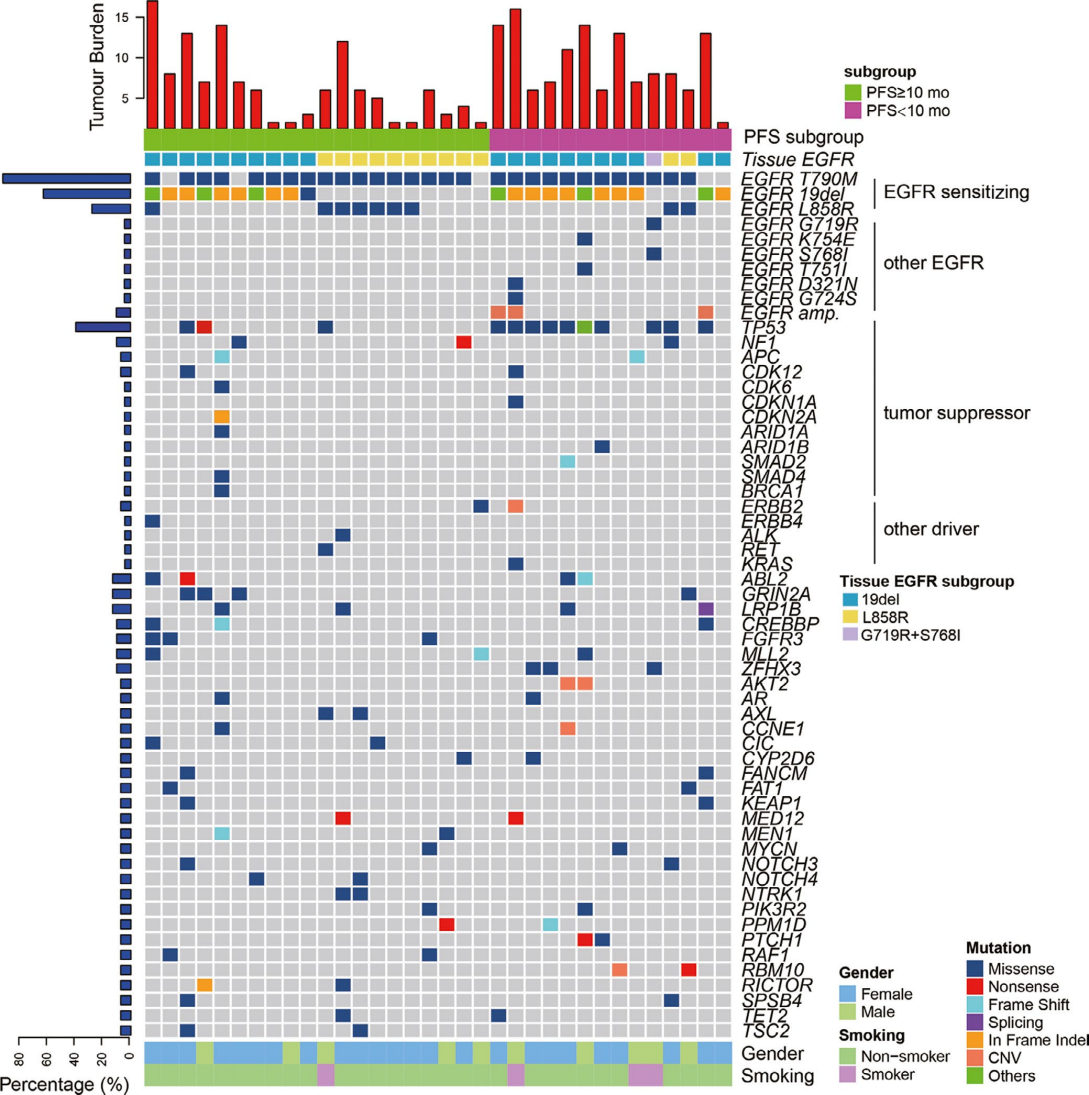
Somatic mutation profiles of 34 patients from pre‐treatment ctDNA. Two patients with negative ctDNA were not shown. Patients were divided into two subgroups by PFS with a cut‐off value of 10 mo. EGFR mutation phenotype previously confirmed by tissue sample was indicated for each patient. Mutation number per Mb region was shown in the upper panel. Genes with somatic mutations occurred in more than one sample were shown in the middle panel. Mutation frequencies of each gene were shown on the left. Gender and smoking status were shown at the bottom. Alteration types are represented by indicated colours

The somatic mutation profiles of the pre‐treatment plasma samples are shown in Figure 1 (n = 34, 2 patients who were ctDNA‐negative were not presented in the figure). Twenty‐nine of the 36 patients (72.2%) had detectable T790M in plasma. A high concordance was also observed for the common EGFR‐sensitizing mutations between pre‐treatment plasma and tissue samples: 30 of the 36 patients were positive, with 21 (58%) 19del, 9 (25%) L858R and 1 (2.7%) S768I. The ctDNA sensitivities of these three genotypes were 88% (21/24), 82% (9/11) and 100% (1/1), respectively.

To better characterize the co‐occurring genetic prognostic features, the patients were divided into two subgroups according to a PFS cut‐off value of 10 months (Figure 1). Besides EGFR‐sensitizing mutations, other EGFR mutations, including G719R, K754E, S768I and T751I, D321N and G724S were detected in 6 patients. EGFR amplification was also detected in 3 patients. Other than EGFR, TP53 is the most frequently mutated gene with 36.1% (13/36) of patients identified TP53‐positive (Figure S1). 76.9% (10/13) of patients belonged to the shorter PFS (<10 months) subgroup. Other concomitant tumour suppressor gene alterations occurred in NF1, APC, CDK6, CDK12, CDKN1A, CDKN2A, ARID1A, ARID1B, SMAD2, SMAD4 and BRAC1 in 9 patients. Other driver genes ERBB2, ERBB4, ALK, RET and KRAS were identified in only one patient. Interestingly, all the novel EGFR mutations, amplification and most (10 out of 13) TP53 mutations were found in the shorter PFS (<10 months) subgroup. This result encouraged a further analysis for the correlation between pre‐treatment ctDNA features and prognosis.

**Figure 2:**
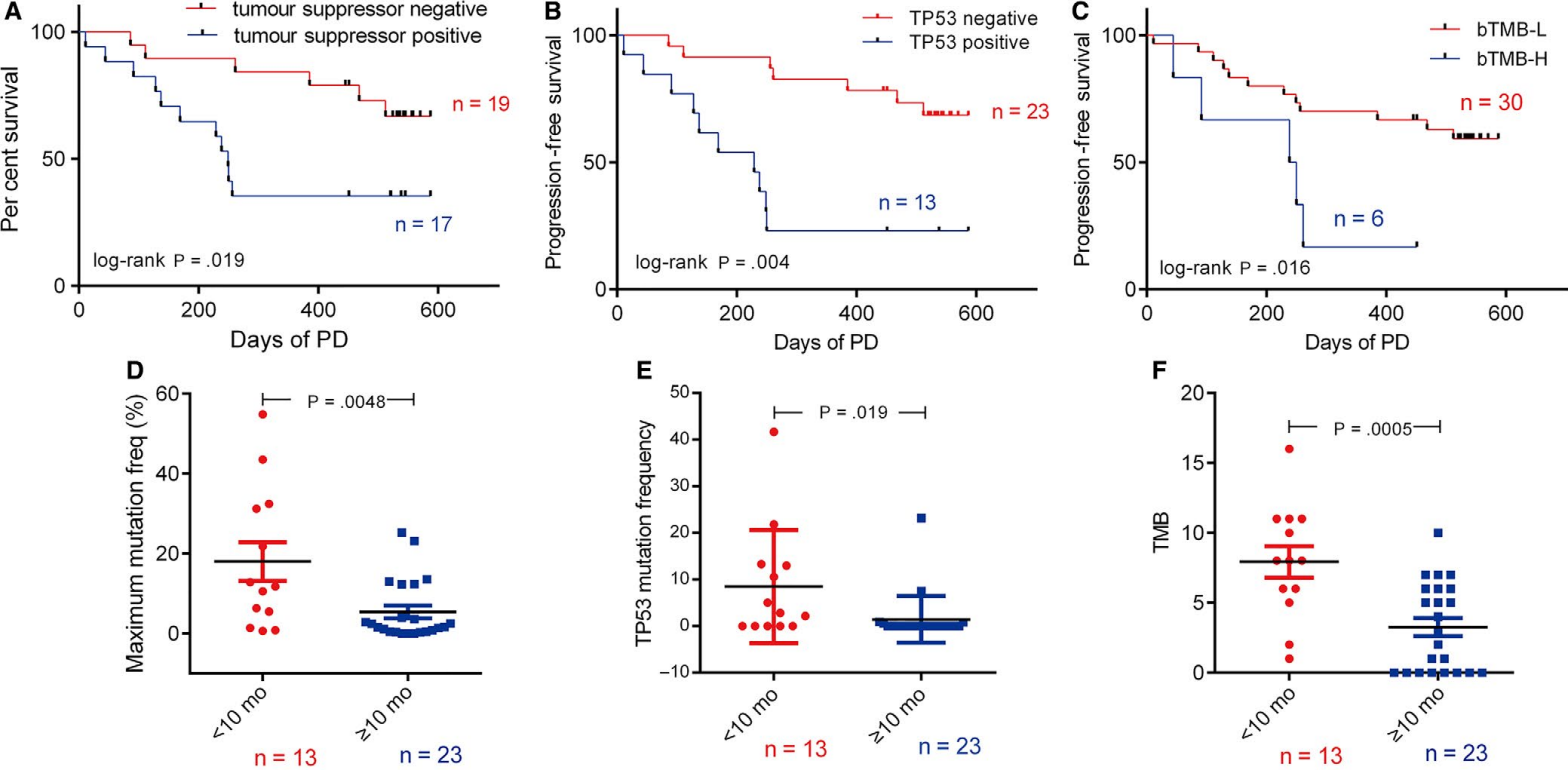
The association between pre‐treatment ctDNA features and PFS in 36 NSCLC patients. A, Progression‐free survival according to baseline tumour suppressor gene in ctDNA (n = 36) [HR 3.05, 95% CI, 1.22‐8.92]. B, Progression‐free survival according to baseline de novo TP53 status in ctDNA (n = 36) [HR 4.62, 95% CI, 2.52‐23.54]. C, Progression‐free survival according to TMB in baseline ctDNA (n‐36) [HR 0.30, 95% CI, 0.034‐0.69]. Comparison of maximum mutation frequency (D), TP53 mutation frequency (C) and TMB (D) between shorter PFS (<10 mo) and longer PFS (≥10 mo) groups. *P* values were determined by log‐rank test and indicated accordingly

We then explored the PFS in patient subgroups divided by EGFR L858R/19del genotype, T790M, TP53 status and additional drivers. The patients with 19del, T790M‐positive, T790M‐subclonal and the subgroup negative for additional driver gene mutations a shorter median PFS than the other subgroups (Figure S2), but the differences were not statistically significant (all *P* > .05). However, patients with suppressor gene mutations or TP53 mutations alone had significantly worse outcomes than those without these mutations (*P* = .04; Figure 2A,B). The median PFS was 313 days (95% CI 212‐415) and 254 days (95% CI 147.8‐361.0) for the suppressor gene and TP53‐positive subgroups, respectively, and was obviously longer (not reached) for the negative subgroups. Specifically, the maximum mutation frequency and TP53 mutation frequency are significantly higher in the shorter PFS subgroup (*P* = .019; Figure 2D,E).

Herein we analysed the correlation between bTMB and the prognosis. Of interest, the bTMB‐H subgroup showed a significantly shorter PFS (244 days) than the bTMB‐L subgroup (*P* = .016; Figure 2C). The patients in shorter PFS group had a median bTMB value of 7.9 mutations/Mb, significantly higher than those in longer PFS group (bTMB = 3.2; Figure 2F). These results suggest that TP53 and the bTMB in pre‐treatment plasma samples were two potentially clinically useful prognostic features in osimertinib treatment patients.

### 3.3 Therapeutic response monitoring using serial ctDNA

**Figure 3:**
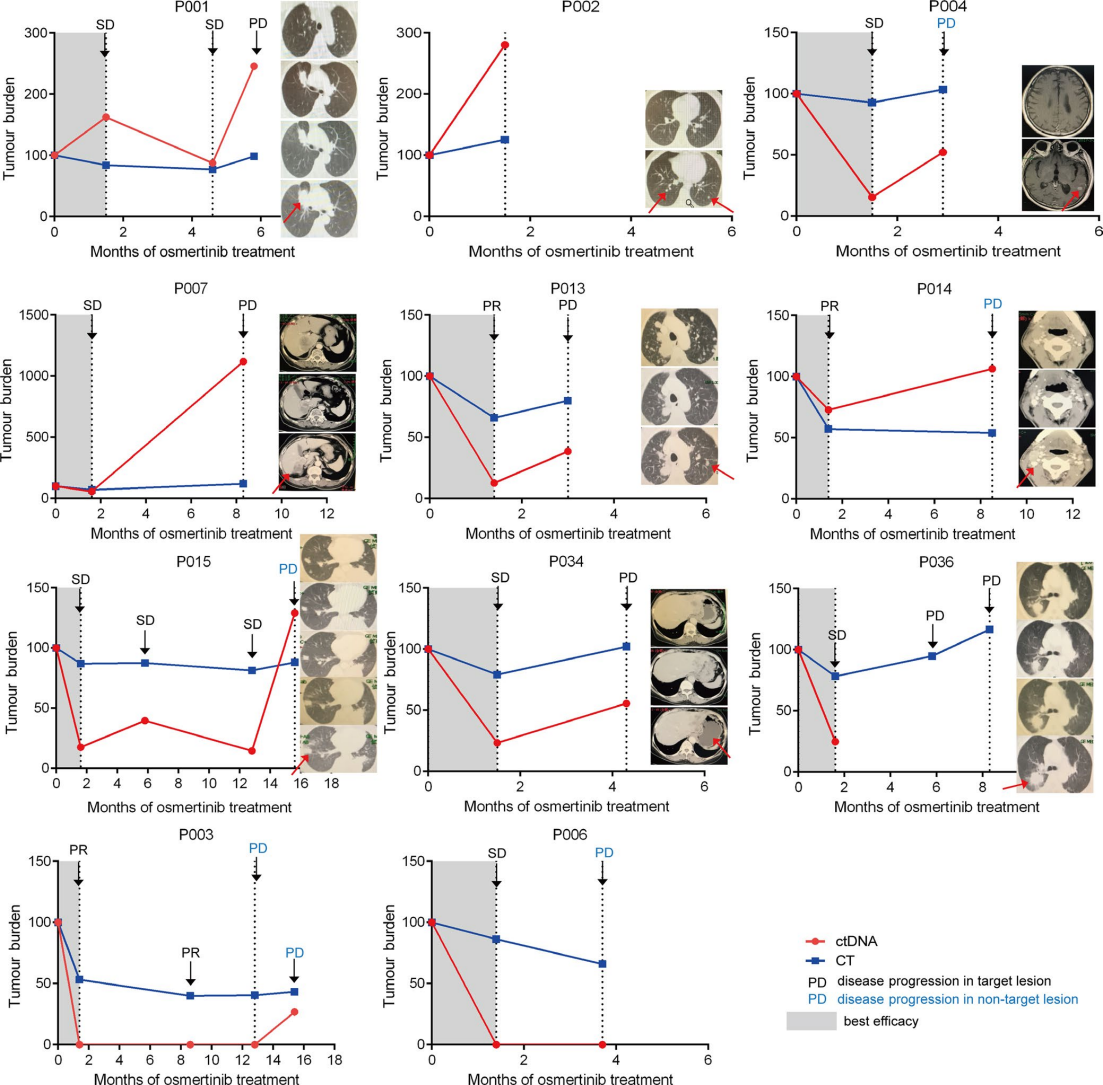
Changes of ctDNA level and sum of longest diameters of target lesions measured by CT. Best efficacy periods are denoted by grey shading. Treatment outcome assessed by RECIST criteria v.1.1 (PR: partial response; SD: stable disease; PD: progressive disease) as ascertained by CT scan at different time‐points is marked by arrows. Patients suffered disease progression at non‐target lesion were marked by blue characters. CT images at each time‐point are shown in a longitudinal direction, from top to bottom. For P004, P007, P014 and P034, metastasis lesions were shown, while for other patients, CT images for target lesions were used

Twelve patients experienced disease progression, 3 of whom had PR and 9 had stable disease (SD) at the first response assessment. Eleven of these 12 patients had pre‐treatment ctDNA detectable at baseline. In order to analyse the correlation between the change of ctDNA level and the change of tumour size in the patients, we defined the difference between the frequency of the ctDNA gene mutation with the highest variant frequency in the trunk clones as tumour burden change in ctDNA. A comparison between ctDNA and response assessment in the 11 PD patients with positive pre‐treatment ctDNA is shown in Figure 3. The ctDNA levels decreased consistent with their tumour shrinkage evaluated in the first response assessment. At disease progression, a rise in ctDNA levels was observed in nine patients, except for one patient without dynamic ctDNA data available. Thus, ctDNA changes correlated with treatment responses in most (90%) patients. It is worth noting that for 3 patients who suffered progression in non‐target disease alone, their increased ctDNA level clearly reflected a PD event (Figure 3). ctDNA failed to reflect disease progression in a non‐target lesion in one patient, which may due to the small tumour size (Figure S3) and subsequent low ctDNA infiltration rate.

### 3.4 Resistance mechanism in twelve progressed patients indicated by paired ctDNA

**Figure 4:**
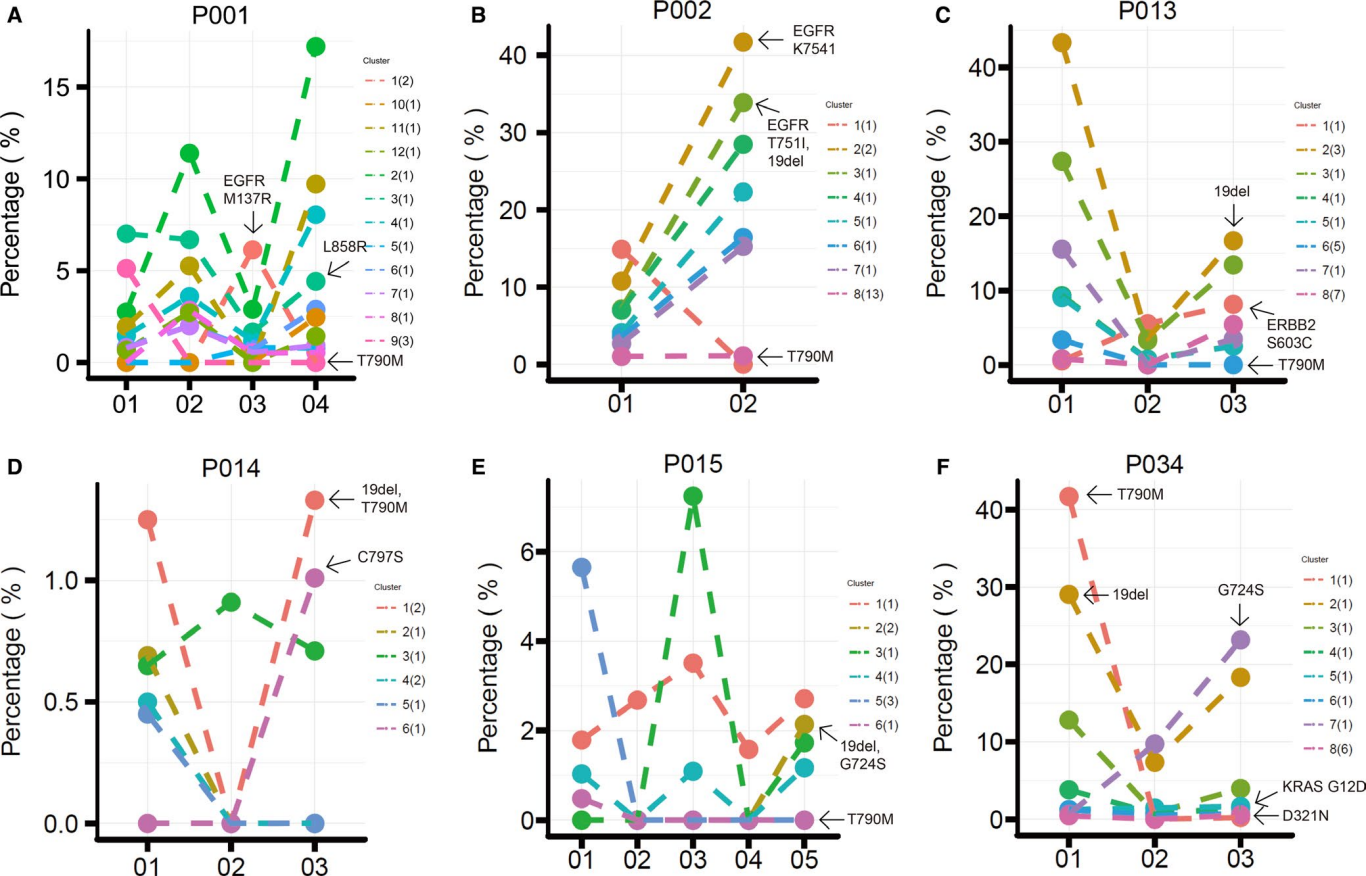
Clonality analysis at efficacy assessment time‐points. Clusters are indicated in different colours, and the mutation numbers included in each cluster are presented in brackets. For each patient, cluster 1 refers to the clonal cluster, the corresponding mutations are trunk mutations; other clusters and mutations are subclonal and branch mutations. The fraction changes of sensitive EGFR mutations, T790M and potential resistant mutations are marked by arrows

**Figure 5:**
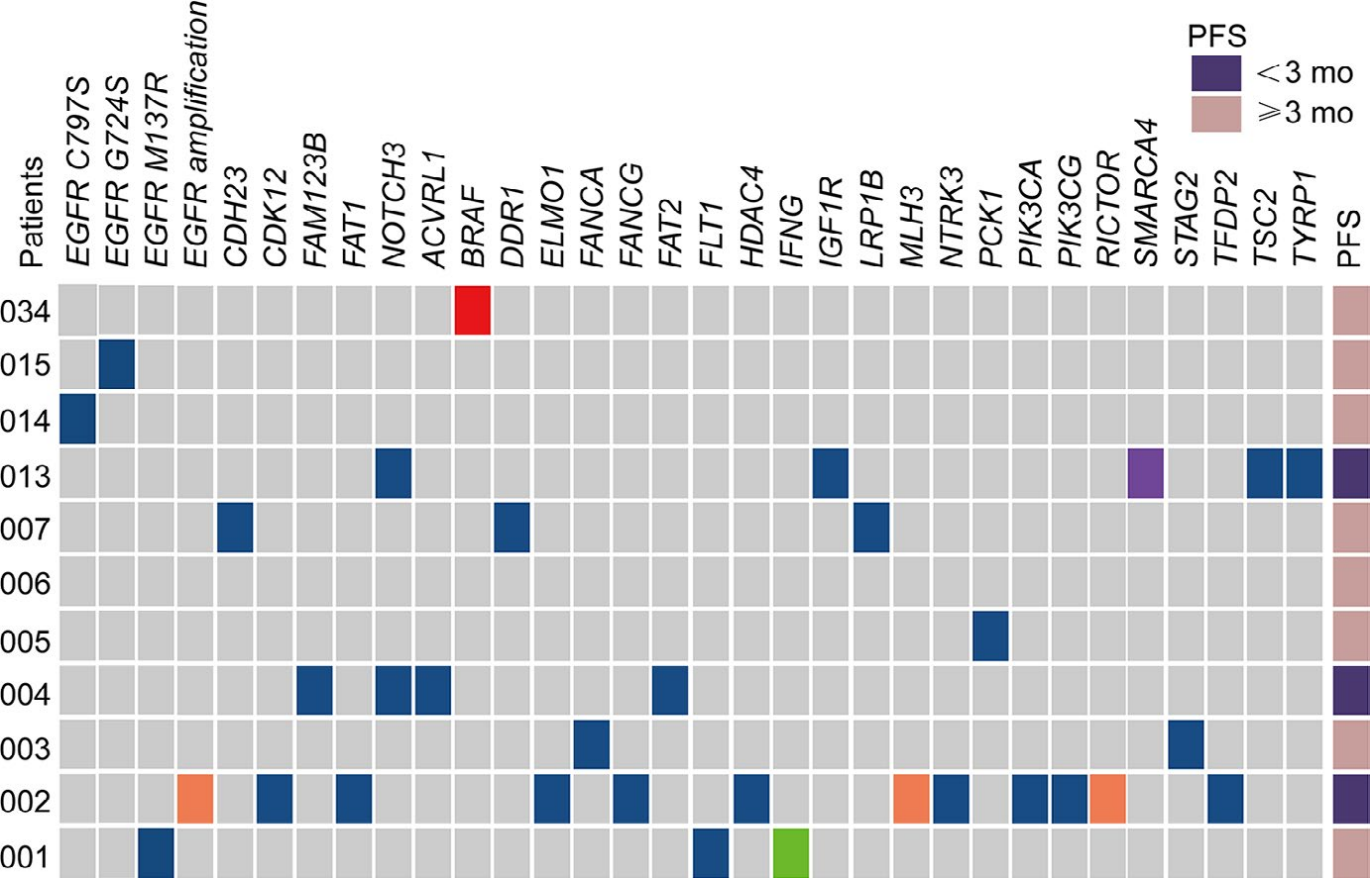
Acquired mutations during treatment in eleven patients who have both positive pre‐treatment and positive after treatment ctDNA results. P036 is not shown due to the lack of after treatment ctDNA data. Alteration types are represented by the same colours as listed in Figure 1. Patients suffered super progression, defined by a <3 mo PFS, are indicated in dark purple, otherwise in pink

Nine of the 12 progressed patients (75%) harboured EGFR T790M mutation in their pre‐treatment ctDNA. Only one patient maintained the T790M in ctDNA at the time of PD, with the emergence of a concomitant C797S mutation. The loss of T790M at resistance may therefore contribute to drug resistance. In addition to T790M loss, other possible resistance mechanisms were identified in 7 patients. The dynamic change of different mutant clusters of the six patients who had more than two ctDNA time‐points during treatment is shown in Figure 4. Among them, four were found to have potential resistant mutations in pre‐treatment samples. Two novel EGFR mutations, K754I and T751I, were detected along with 19del in pre‐treatment ctDNA (n = 1). Upon PD, T790M was completely lost while K754I and T751I clones were dramatically increased. PIK3CA E542K was also found at PD (n = 1), although the allele frequency was as low as 0.49%. Besides ERBB2 amplification, a novel ERBB2 S603C mutation was also detected in the baseline sample (n = 1). The variant frequency of the S603C clone gradually increased during treatment, while the other clones decreased compared to the baseline, suggesting a possible dominate resistance role of the S603C subclone. Three potential resistance mutations co‐existed in one patient's pre‐treatment ctDNA: G724S, EGFR and ERBB2 amplification. The G724S clone sharply increased along with 19del after an initial PR, which may explain the early resistance at 4.5 months. KRAS K12D and a novel EGFR D321N mutation were also found (n = 1). Two patients found to have novel acquired resistance mutations at the time of PD. One had a novel EGFR mutation M137R detected, with the osimertinib sensitive clone containing L858R and T790M decreased. The other acquired a tertiary mutation, C797S, in‐cis with T790M. The transient benefit of osimertinib in one patient may be due to subclonal T790M and the emergence of another clone with G724S. All acquired mutations at PD are presented in Figure 5. The three patients who had PFS < 3 months had more acquired mutations than others (Figure S4).

**Figure 6:**
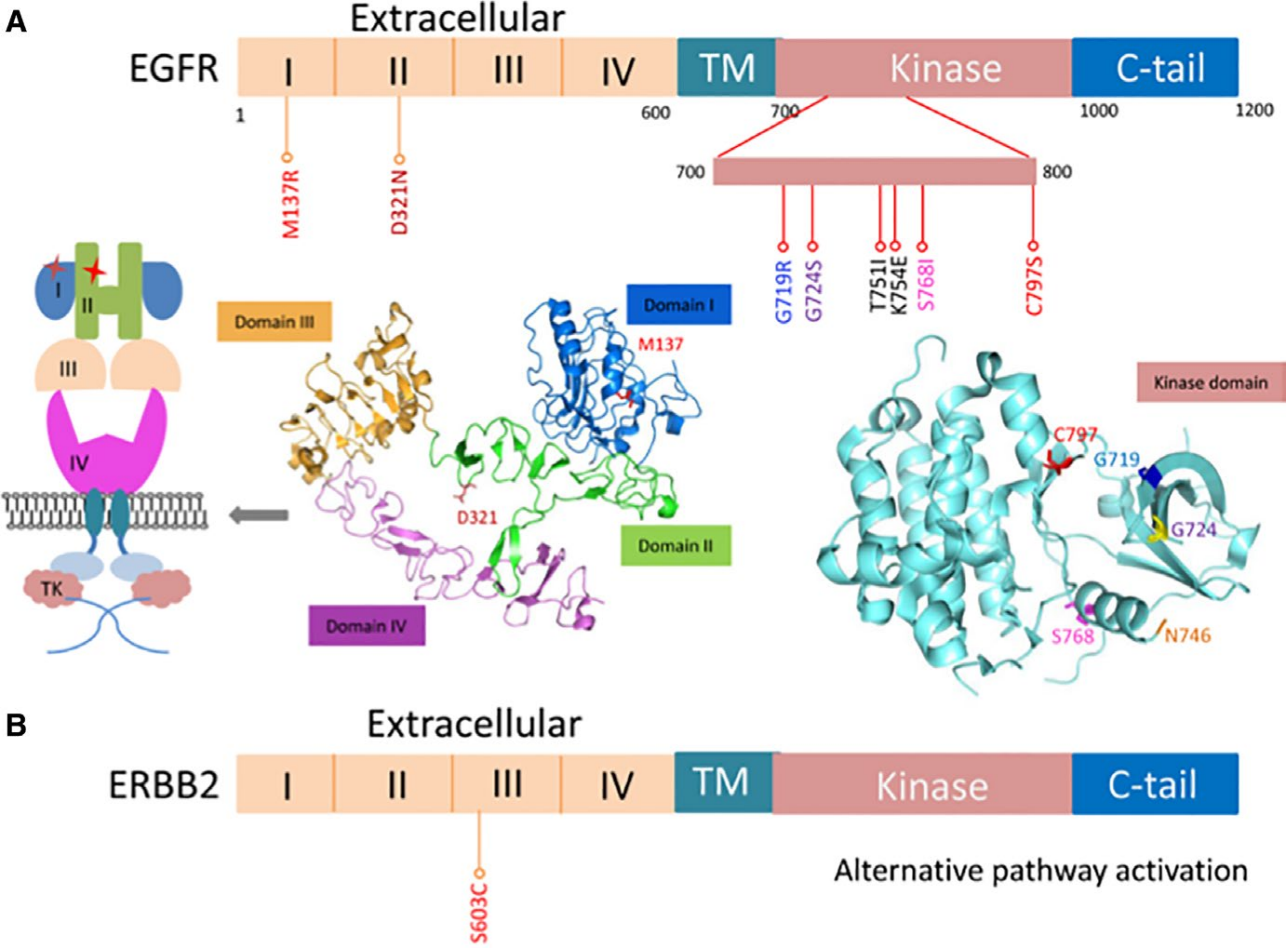
Positions of the EGFR (A) and ERBB2 (B) mutations identified in PD patients. The domain organizations of EGFR are shown with site mutations annotated by sticks. The crystal structure of wild‐type EGFR extracellular domains and kinase domain is presented. Four extracellular domains are shown in different colours. A schematic model for the two extracellular mutations is also presented

All EGFR family mutations found in the 12 progressed patients are annotated in schematic models in Figure 6. Six of these EGFR mutations caused amino acid change in kinase domain, and the other 2 occurred in extracellular domain. T751I and K754E are located adjacent to the exon 19 deletion region (E746‐A750). Structurally, these two amino acids belong to the flexible loop region where the local crystal structure was not available (Figure 6A), and N746 was marked in orange in the figure to show the end of the visible alpha‐helix. We speculated that these two mutations may affect the binding mode of osimertinib to the EGFR TK domain and cause a lower sensitivity to the small molecule inhibitor, although further function studies are needed to draw any conclusions. M137R and D321N were located in domain I and domain II of the extracellular domain, which might affect dimerization and the subsequent activation activity. Moreover, ERBB2 S603C was also located in subdomain IV of the extracellular domain (Figure 5B) of this EGFR family protein.

## 4 DISCUSSION

Compared to tissue biopsy, liquid biopsy, especially ctDNA sequencing, allows comprehensive and longitudinal monitoring of tumour genomics non‐invasively. While the correlation of ctDNA and efficacy of EGFR‐TKI has been extensively investigated,^21^ this work presents one of the most comprehensive ctDNA genomic analyses of tumour evolution during osimertinib treatment, which is a third‐generation EGFR‐TKI. The present study demonstrated that ctDNA is an important approach to predict prognosis and monitor response.

Concurrent mutations, especially TP53, KRAS and PIK3CA, are common in EGFR‐mutated NSCLC. Coexisting TP53 mutations were generally associated with shorter survival in first‐generation EGFR‐TKI therapy.^14^, ^22^ In our study, TP53 mutations and tumour suppressor genes strongly correlated with a worse outcome during osimertinib treatment. It is worth noting that one particular patient harbouring multiple other suppressor gene mutations without TP53 mutation had a PFS ≥ 10 months.

T790M is another ctDNA feature extensively discussed as a prognostic marker in osimertinib treatment. The sensitivity of pre‐treatment ctDNA T790M is 73% in our cohort with documented tissue T790M which is similar to previous studies.^23^ Possible explanations for the lower sensitivity of ctDNA compared to tissue DNA include the tumour size, location, shedding characteristics and degradation during handling of samples. Specifically, several studies have demonstrated the spatial heterogeneity of T790M. However, either T790M alone or T790M/sensitizing mutation ratio failed to consistently predict the outcome from osimertinib,^21^, ^24^, ^25^ indicating a complex clinical interpretation for T790M status. Similarly, in our study, T790M existence and clonal status in plasma failed to identify two subgroups with significantly different PFS, although patients who lost detectable T790M during treatment were more likely to develop early resistance and have a shorter PFS.

Tissue‐based TMB (tTMB) is an emerging biomarker of response to checkpoint inhibitor treatment.^26^, ^27^ Recent studies have reported the utility of bTMB in predicting the clinical outcomes.^6^ In EGFR‐TKI treatment, TMB is rarely discussed since tumours with EGFR sensitive mutation usually have a low TMB. Consistent with this general concept, only 6 patients were TMB‐H in our cohort, using a cut‐off value selected from our own NSCLC database using this platform and there was a significant difference on PFS between bTMB‐H and bTMB‐L subgroups. With the intriguing observation of unexpectedly good outcomes with the atezolizumab/bevacizumab combination in EGFR‐mutant patients in IMPower 150, TMB could emerge as an important marker in this subgroup.

Besides monitoring therapeutic response and clonality evolution,^28^, ^29^, ^30^, ^31^, ^32^ ctDNA has been indicated to detect the development of tumour heterogeneity, providing earlier detection of disease progression or recurrence. In our study, 5 patients with progression in non‐target lesions showed obvious increases of ctDNA. Four of these five patients had brain or liver metastases, suggesting that a clinically significant signal of emerging lesions could be detected by ctDNA. ctDNA can detect small insidious lesions which cannot be detected with routine imaging, possibly because it can draw a whole picture of the tumour burden. However, for patients with small lesion or low level of ctDNA, the ability of ctDNA to track progression is precluded to some extent. We did not see earlier ctDNA detection of progression before CT‐based assessment of disease progression as previously reported, which may due to a less frequent ctDNA sampling time‐points in this study but suggests that early detection of progression by ctDNA may not be clinically significant.

In spite of several mechanisms identified correlated with resistance, the complex resistance mechanism is not obvious. ctDNA pre‐treatment and at PD provide hints for better understanding the resistant mechanisms such as EGFR C797S, G724S, G719R, KRAS G12D, PIK3CA E542K, EGFR amplification and ERBB2 amplification. Other potential EGFR‐dependent resistance mechanisms were identified, including EGFR T751I and K754E in the kinase domain, and D321N and M137R in extracellular domain. It is difficult to perform a docking simulation for the binding of EGFR T751I/K754E and osimertinib because of the unavailability of local structure around A750.^33^ Although rarely reported, some EGFR extracellular domain mutations have been correlated with resistance to EGFR‐TKI.^34^ Further functional experiments would help to investigate binding mode changes of the TK domain mutants and EGFR activity changes as a consequence of the two extracellular mutants. Despite extensive structural homology of the extracellular putative ligand binding region, ERBB2 has no identified direct ligand. Instead, it acts as the preferred dimerization partner for all other ERBB family receptors.^35^ A novel mutation ERBB2 S603C was discovered located in the extracellular domain. We speculated that the new cysteine in subdomain IV of extracellular domain may lead into improper dimerization between ERBB family members and cause alternative pathway activation.

## 5 CONCLUSIONS

Our study explored the potential applications of serial ctDNA analysis in the management of advanced lung adenocarcinoma patients. ctDNA could help with clinical practice during osimertinib treatment, regarding monitoring tumour response, detecting development of heterogeneity, identifying potential resistance mechanisms, predicting treatment efficacy and patient outcome.

## CONFLICT OF INTEREST

Sha Wang, Lianpeng Chang, Yanfang Guan and Xin Yi are current employees of Geneplus‐Beijing. All other authors declare no competing interest.

## AUTHORS' CONTRIBUTIONS

YKS contributed to study design and conception, data analysis and interpretation. All authors were involved in data acquisition. All authors had full access to all the date in the study and contributed to the writing of the report, reviewed it for intellectual content and approved the submitted version.

## Data Availability

Some information was provided in the supplementary files, and all other relevant data could be obtained from the corresponding authors of this study.
