# Supplementary material for: Co‐mutational assessment of circulating tumour DNA (ctDNA) during osimertinib treatment for T790M mutant lung cancer
Source: J Cell Mol Med. 2019 Aug 8;23(10):6812–21. doi: 10.1111/jcmm.14565 (PMC6787503; doi:10.1111/jcmm.14565)
Supplement: Supplementary file 1 [file JCMM-23-6812-s001.docx]

**Supplementary Figures of “Co-mutational assessment of circulating tumor DNA (ctDNA) during osimertinib treatment for T790M mutant lung cancer”**


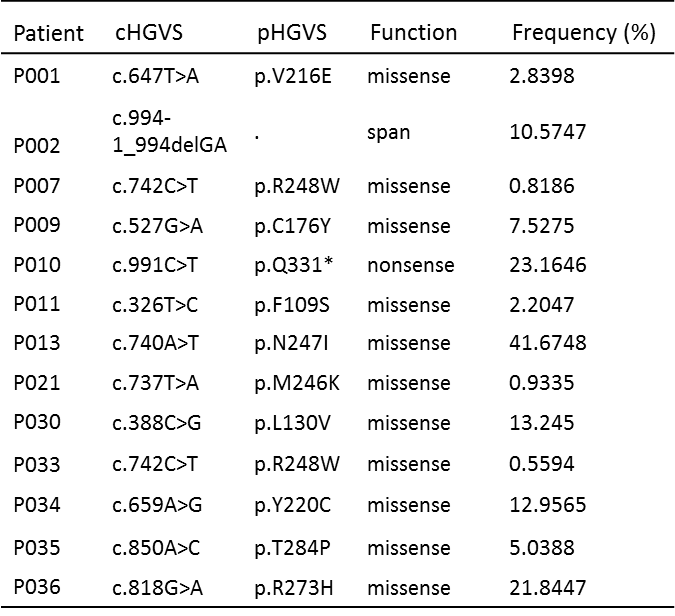


**Sfig 1.** Mutation of TP53 detected in patients in this study.


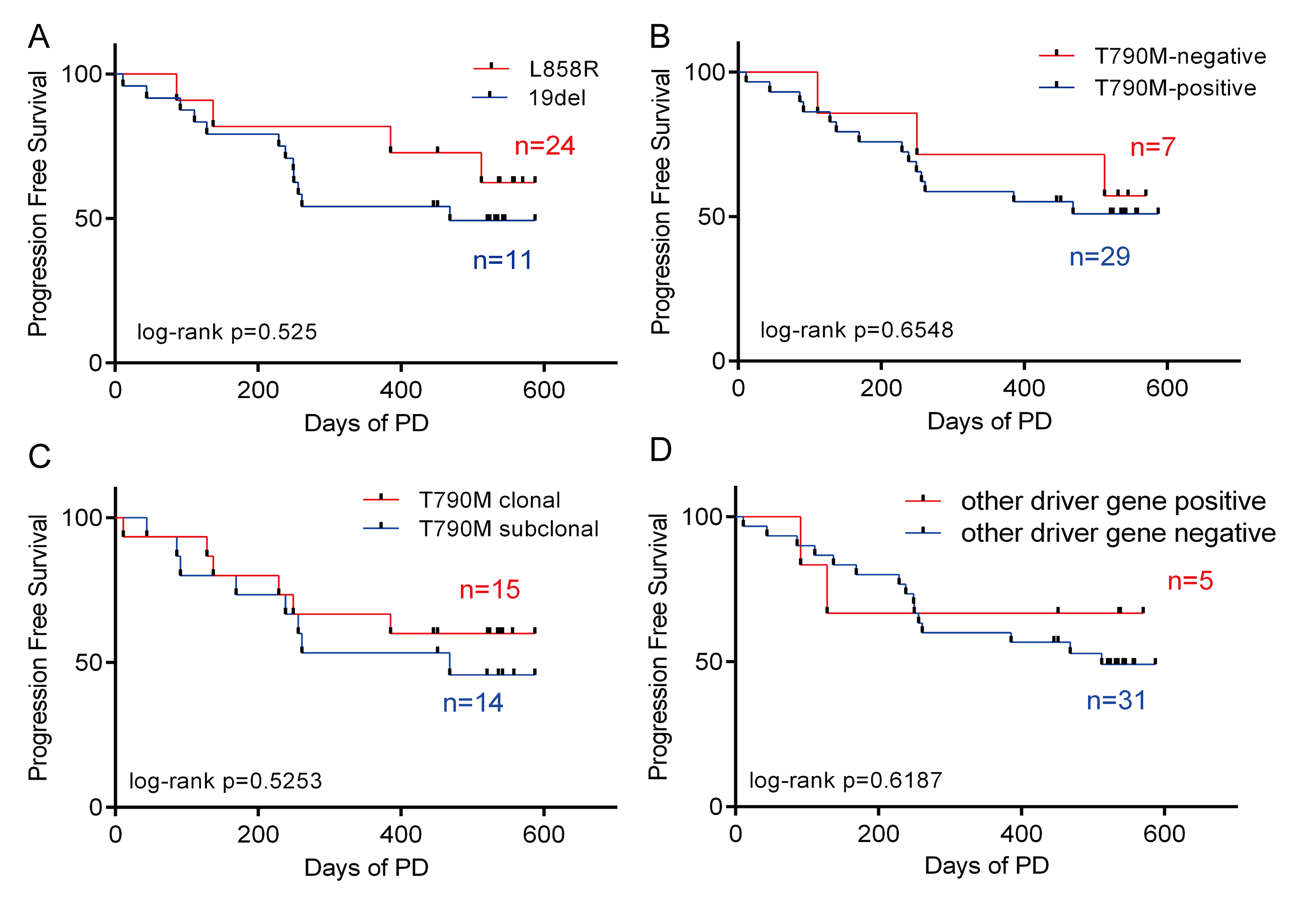


**Sfig2.** The association of the mutation status of EGFR L858R/19del genotype, T790M, TP53 status and additional drivers with PFS of patients treated with Osimertinib.


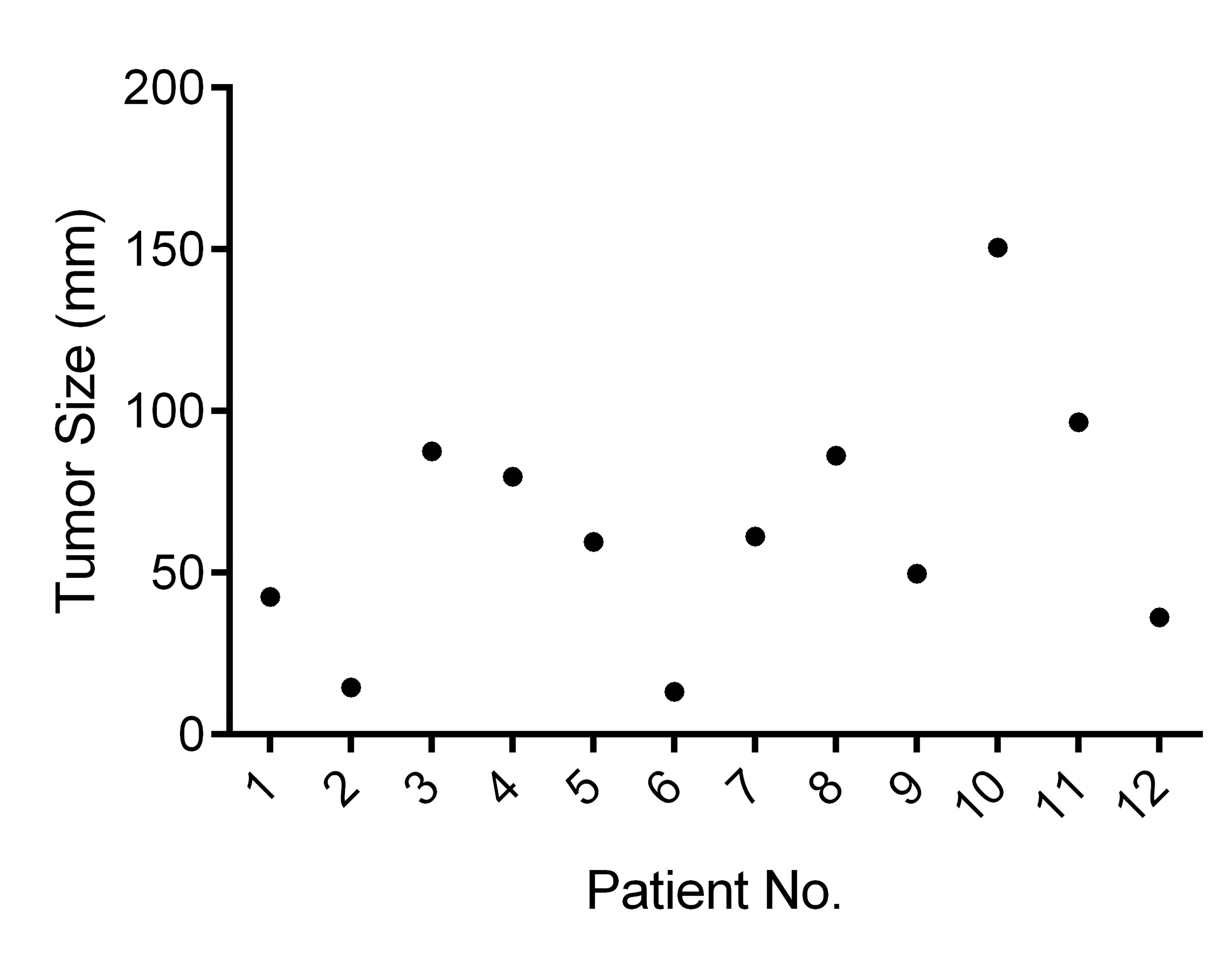


**Sfig3.** The target lesion tumor size of 12 patients experienced disease progression.


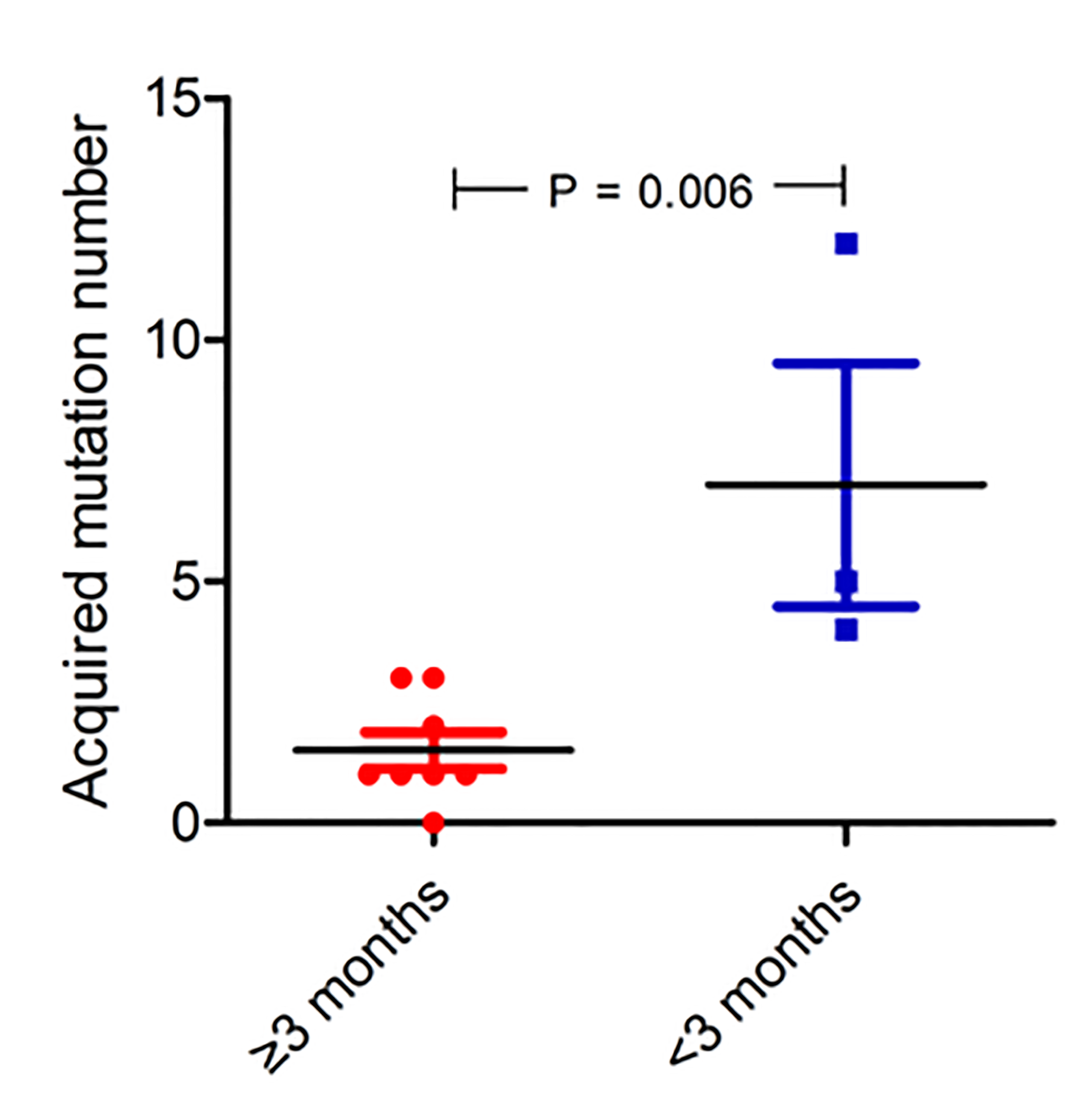


**Sfig4.** The number of acquired mutations in patients who had PFS＜3 months and PFS ≥ 3 months.
